# Preclinical Absorption, Distribution, Metabolism, and Excretion of Sodium Danshensu, One of the Main Water-Soluble Ingredients in *Salvia miltiorrhiza*, in Rats

**DOI:** 10.3389/fphar.2019.00554

**Published:** 2019-05-29

**Authors:** Xiangguo Meng, Jingjing Jiang, Hui Pan, Shengyuan Wu, Shuowen Wang, Yuefen Lou, Guorong Fan

**Affiliations:** ^1^ Department of Pharmacy, Shanghai University of Medicine and Health Sciences, Shanghai, China; ^2^ Department of Pharmacy, Shanghai Fourth People’s Hospital, Shanghai, China; ^3^ Department of Clinical Pharmacy, Shanghai General Hospital, School of Medicine, Shanghai Jiao Tong University, Shanghai, China; ^4^ Laboratory of Drug Metabolism and Pharmacokinetics, School of Medicine, Tongji University, Shanghai, China; ^5^ Shanghai Key Laboratory for Pharmaceutical Metabolite Research, School of Pharmacy, Second Military Medical University, Shanghai, China

**Keywords:** sodium danshensu, pharmacokinetics, LC-MS/MS, ADME, bioavailability study

## Abstract

In this study, the absorption, distribution, metabolism and excretion (ADME) of sodium danshensu (Sodium DL-β-(3, 4-dihydroxyphenyl)lactate), one of the main water-soluble active constituents in *Salvia miltiorrhiza*, were evaluated in rats. Pharmacokinetic study was evaluated in doses of 15, 30, and 60 mg/kg after intravenous administration of sodium danshensu. Bioavailability study was evaluated by comparing between 30 mg/kg (I.V.) and 180 mg/kg (P.O.) of sodium danshensu. Tissue distribution, metabolism, and excretion were evaluated at 30 mg/kg (I.V.) of sodium danshensu. Following intravenous administration, sodium danshensu exhibited linear pharmacokinetics in the dose range of 15–60 mg/kg. Sodium danshensu appeared to be poorly absorbed after oral administration, with an absolute bioavailability of 13.72%. The primary distribution tissue was kidney, but it was also distributed to lung, stomach, muscle, uterus, heart, etc. Within 96 h after intravenous administration, 46.99% was excreted *via* urine and 1.16% was excreted *via* feces as the parent drug. Biliary excretion of sodium danshensu was about 0.83% for 24 h. Metabolites in urine were identified as methylation, sulfation, both methylation and sulfation, and acetylation of danshensu. Sodium danshensu can be developed as an injection because of its poor oral bioavailability. In conclusion, sodium danshensu is widely distributed, mainly phase II metabolized and excreted primarily in urine as an unchanged drug in rats.

## Background

*Salvia miltiorrhiza*, named Danshen in Chinese, is one of the most versatile traditional Chinese medicinal herbs. It has been widely used to treat and prevent cardio vascular disease, hyperlipidemia and cerebro vascular disease throughout the world (Cheng 2007; Wang et al., 2017). Up to today, more than 70 compounds have been isolated and a wide spectrum of secondary metabolites has been identified from *Salvia miltiorrhiza*, including diterpenoid quinines, hydrophilic phenolic acids and essential oil constituents (Li et al., 2009). It is generally known that lipophilic diterpenoid quinines and hydrophilic phenolic acids are the main bioactive components in *Salvia miltiorrhiza* (Su et al., 2015).

Danshensu, also named as salvinanic acid, is the main water-soluble phenolic acids in *Salvia miltiorrhiza*. It has been reported that danshensu exhibited several pharmacological activities such as cardioprotective effect by inhibiting L-type calcium channels (Song et al., 2016), radioprotective effect by scavenging reactive oxygen species (Guo et al., 2013), protection of vascular endothelial cells by an antioxidative mechanism (Zhao et al., 2012), protection on liver injured by an antioxidative mechanism (Wang et al., 2007), etc.

**Figure 1:**
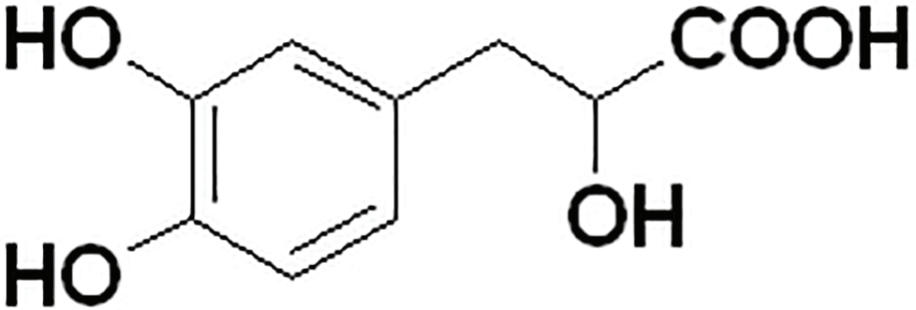
Chemical structure of (D/L) sodium danshensu.

Because danshensu is unstable in the nature, it was transformed into its sodium salt, sodium danshensu (sodium DL-β-(3,4-dihydroxyphenyl)lactate, Figure 1). Sodium danshensu has the same effectiveness as danshensu (Wang and Xu, 2005; Wang et al., 2007; Zhao et al., 2013; Jia et al., 2018). Due to its potential pharmacological activities, sodium danshensu has attracted more and more attention recently and is being investigated as a new drug (Zhou et al., 2009; Wei et al., 2018). In order to investigate the pharmacokinetics of sodium danshensu, several biological analytical methods have been reported (Li et al., 2008; Zhan et al., 2008; Liu et al., 2010, 2014; Fu et al., 2014; Zheng et al., 2015), and a high-throughput, simple, sensitive, selective and reliable LC-MS/MS method was developed and validated in our laboratory before (Jiang et al., 2017). Although many researchers published several articles about pharmacokinetic study of sodium danshensu in rats, detailed pharmacokinetic characteristics including absorption, distribution, metabolism, and elimination (ADME) of sodium danshensu have not been reported. In this study, we extensively investigated the preclinical ADME and bioavailability study of sodium danshensu in rats.

## Materials and Methods

### Chemicals and Reagents

Sodium danshensu (purity 98.5%) was made and provided by the Prof. Chuan Zhang of School of Pharmacy, Second Military Medical University, Shanghai (Zhang et al., 2010). Ketoprofen (purity ≥95%), the internal standards (IS), was purchased from the National Institute for Food and Drug Control (Beijing, China). HPLC-grade methanol and acetonitrile were purchased from Merck (Darmstadt, Germany). Ethyl acetate was obtained from Shanghai Sheng De Chemical Co., Ltd. (Shanghai, China). Formic acid was HPLC grade and purchased from TEDIA Company (Fairfield, CA, USA). Deionized water was prepared by using the Milli-Q Plus Ultrapure Water System (Millipore Corporation, Bedford, MA). All other chemicals used in this study were of the highest quality available.

### Animals

Sprague–Dawley (SD) rats (male and female equally, 180–220 kg, 6–8 weeks) were purchased from the Shanghai Slack Experimental Animal co., LTD (Shanghai, China) and were acclimated in laboratory for at least 1 week prior to the study. The animal room was controlled to maintain a temperature of 18–25°C and relative humidity of 20–60%. The animals were housed under a 12-h light/dark cycles and allowed free access to food and water.

### Pharmacokinetic Study

Rats were fasted overnight before dosing and 6 h after dosing. For the pharmacokinetic study and bioavailability study, 24 rats were randomly divided into four groups (*n* = 24, 6 per group) and were respectively designed as different dosages (15, 30, 60 mg/kg I.V., 180 mg/kg P.O.).

Blood samples were serially collected from orbital venous plexus into heparinized tubes pre-dose and at 0, 0.08, 0.17, 0.33, 0.67, 1.0, 1.5, 2.0, 3.0, 4.0, 6.0, 8.0, and 12.0 h after dosing for the three intravenous injection groups. For the oral administration group, blood samples were serially collected from orbital venous plexus into heparinized tubes pre-dose and at 0.08, 0.17, 0.25, 0.5, 0.75, 1.0, 1.5, 2.0, 3.0, 4.0, 6.0, 8.0, and 12.0 h after dosing. All rats were transfused normal saline after drawing blood. Plasma was then separated from blood by centrifuging at 3,500 rpm for 10 min immediately and transferred to another tubes and stored at −80°C before analysis.

### Distribution

In tissue distribution study, rats (*n* = 36, 6 per group) were randomly divided into six groups and sex distribution equally per group, intravenously injected with a single dose of 30 mg/kg sodium danshensu. At 0.17, 0.67, 1, 2, 4, and 6 h after dosing, rats were anesthetized with diethyl ether and sacrificed. Tissues (heart, liver, spleen, lung, kidney, brain, stomach, intestinal, fat, muscle, testis, ovary, and uterus) were excised immediately, rinsed with saline, dried with filter paper, weighed, and homogenized in saline. Tissue homogenates were stored at −80°C before analysis.

### Metabolism

Metabolites were analyzed in rat urine with intravenously injected with a single dose of 30 mg/kg sodium danshensu by LC-MS/MS. An aliquot of 500 μl of urine was acidified with 250 μl (3 mol/L) of HCl and vortexed. The mixture was then loaded to activate C18 SPE cartridges, washed with 1 ml of water, and eluted with 1 ml of 80% methanol. The eluant was then evaporated to dryness under a nitrogen gas stream and reconstituted with 100 μl of mobile phase, and centrifuged at 12,000 rpm for 10 min. The supernatant was then transferred and applied for LC-MS/MS detection.

### Excretion

Urine and feces samples were obtained from rats (*n* = 6) intravenously administrated with intravenously injected with a single dose of 30 mg/kg sodium danshensu and housed individually in metabolic cages equipped with a urine and feces separator. The urine and fecal samples were collected pre-dose and at 0–2, 2–4, 4–6, 6–8, 8–12, 12–24, 24–36, 36–48, 48–60, 60–72, 72–84, and 84–96 h after doing. Urine and feces were stored at −80°C before analysis.

In biliary excretion study, SD rats (*n* = 6) were anesthetized with urethane (1.2 g/kg, ip) and the bile duct was cannulated. After intravenous administration with a single dose of 30 mg/kg sodium danshensu, bile was collected at 2 h intervals for the first 12 h and at 24 h. Bile samples were stored at −80°C before analysis.

### Sample Preparation

In the present study, sodium danshensu and IS were extracted from rat plasma by using a liquid-liquid extraction method. A 25 μl aliquot of IS solution containing 40.16 ng/ml of ketoprofen was added to a 50 μl aliquot of rat plasma and then vortexed and acidified with 20 μl of 3 mol/L HCl. The mixture was then extracted with 1 ml of ethyl acetate, vortexed, and centrifuged at 5,000 rpm for 10 min. A 800 μl aliquot of the supernatant was then transferred to another tube and evaporated to dry. The residue was then reconstituted with 50 μl of mobile phase and centrifuged at 12,000 rpm for 10 min. The supernatant was then transferred to LC-MS/MS detection.

In the tissue distribution study, 50 μl IS solution (52.6 ng/ml) was added to 100 μl tissue homogenates respectively. The mixed samples were vibrated 30 s and acidified with 50 μl of 3 mol/L HCl. The rest of the experimental operation was same as the plasma samples’ process.

In the excretion study, feces were prepared by ultrasonic extraction in 0.9%NS for 5 min and vortex for 3 min. After centrifuging at 3,500 rpm for 10 min, 100 μl supernatant fecal extract was prepared immediately before analysis. The sample preparation for urine, bile and fecal extract were the same to plasma samples. Except that the concentrations of IS or the volumes of HCl were different. A 50 μl aliquot of IS solutions at concentrations of 312, 103.2, and 103.2 ng/ml were added to a 100 μl aliquot of urine, bile, or feces samples, respectively. And all were acidified with 50 μl of 3 mol/L HCl by 30 s.

### LC-MS/MS Conditions

The method combining liquid chromatography with electrospray ionization tandem mass spectrometry to determine the sodium danshensu in plasma was developed and validated as described previously (Yu et al., 2011). Analysis was performed using a VARIAN 1200L HPLC-MS system equipped with VARIAN ProStar 210 pump, VARIAN ProStar 410 autosampler, VARIAN 1200L Quadrupole MS/MS, and VARIAN MS 6.8 workstation. The analytes were separated by a Diamonsil C_18_ column (200 mm × 4.6 mm, 5 μm) at 25°C with a mobile phase consisting of methanol and 0.1% formic acid in water (80:20, v/v) at a flow rate of 0.8 ml/min, and 2/5 of flow was entered into mass spectrometer. Injection volume was set at 20 μl and analysis time was set at 9 min. Electrospray ionization was operated in a negative ion mode. The optimized precursor-to-product ion transitions were monitored at m/z 197–135 and 253–209 for sodium danshensu and IS, respectively. Source-dependent parameters were set as follows: needle −4,700 V; shield −150 V; nebulizing gas flow 3.31 Mpa; drying gas 250°C, 1.38 MPa; capillary voltage −40 V; and collision energy 18.0 and 5.5 V for sodium danshensu and IS.

### Data Analysis

Pharmacokinetic parameters of sodium danshensu were analyzed by using the BAPP software (version 3.1, Center of Drug Metabolism and Pharmacokinetics, China Pharmaceutical University, Nanjing, China) with a noncompartmental model. All data are presented as mean ± SD. The absolute bioavailability (%*F*) of sodium danshensu was estimated *via* the ratio of AUC_0−∞_ after oral and intravenous administration.

## Results

### Pharmacokinetic Results

**Figure 2:**
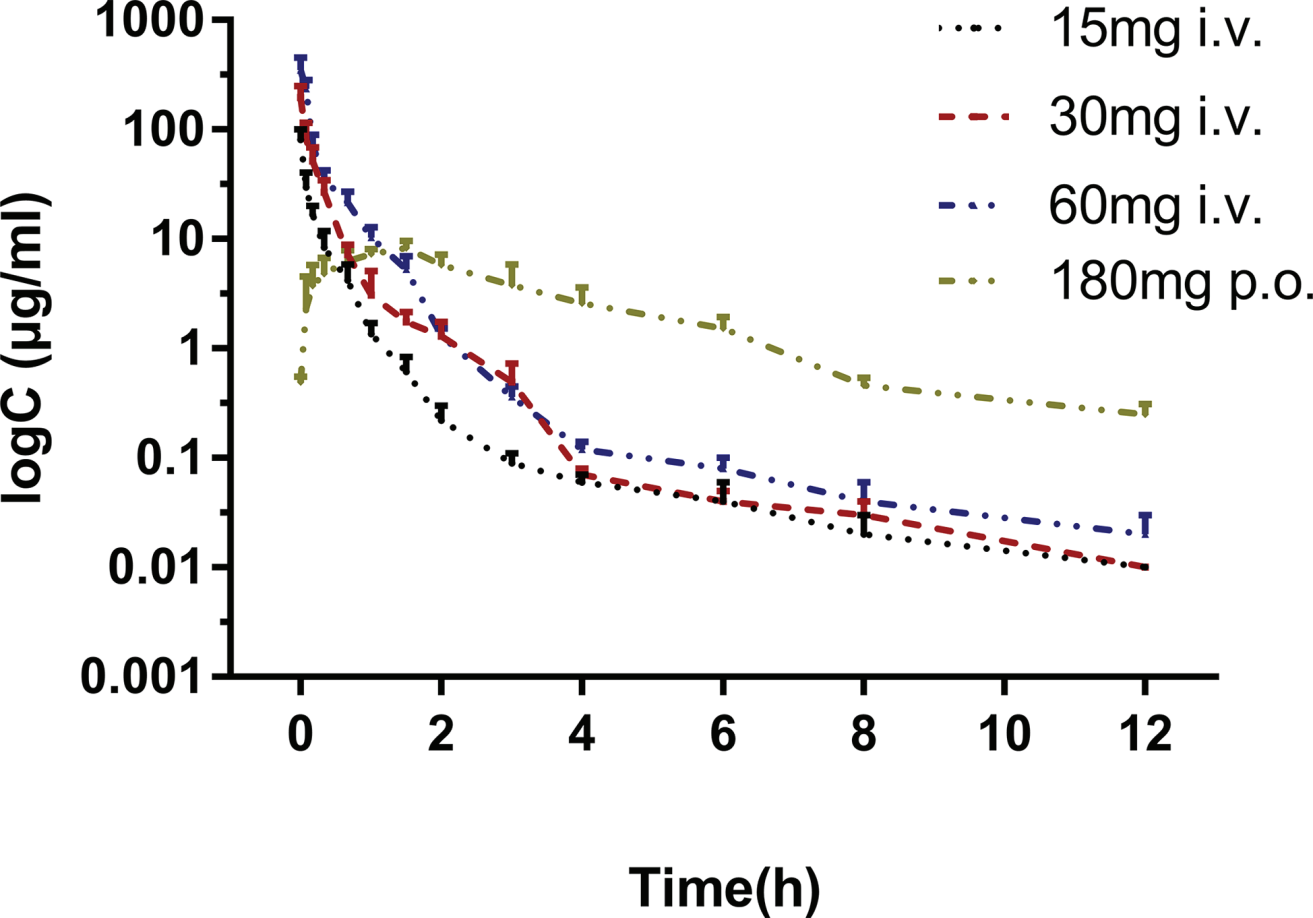
Mean plasma concentration-time curves of sodium danshensu after intravenous administration of 15, 30, 60 mg/kg and oral administration 180 mg/kg in rats (*n* = 6).

The pharmacokinetic profiles of sodium danshensu in rats following intravenous administration of sodium danshensu at doses of 15, 30, and 60 mg/kg are shown in Figure 2, and relevant pharmacokinetic parameters obtained are listed in Table 1. Plasma concentrations of sodium danshensu decreased quickly with elimination half time (*t*_1/2_) of 2.76 ± 0.72, 3.00 ± 0.31, and 2.64 ± 0.44 h, respectively. The AUC_0−12_ values were determined to be 12.67 ± 1.40, 34.27 ± 2.49, and 67.70 ± 11.71 μg h/ml. The results of the variance analysis showed that the t_1/2_ and MRT had no significant differences among different groups (*p* > 0.05). It seems that exposure increases with dosages in the dose range of 15–60 mg/kg and the relationship between AUC_0−12_ and dose was reflected in the liner correlation coefficient (*r*^2^ = 0.9338).

**Table 1:** Pharmacokinetic parameters of sodium danshensu in rats after intravenous administration of 15, 30, and 60 mg/kg and oral of 180 mg/kg (*n* = 6).

Parameters	Units	15 mg/kg (I.V.)	30 mg/kg (I.V.)	60 mg/kg (I.V.)	180 mg/kg (P.O.)
*T*_max_	h	–	–	–	1.40 ± 0.30
*C*_max_	μg/ml	81.18 ± 19.26	195.32 ± 53.15	349.32 ± 104.85	8.76 ± 0.85
*t*_1/2_	h	2.76 ± 0.72	3.00 ± 0.31	2.64 ± 0.44	2.35 ± 0.25
AUC_0−12_	μg h/ml	12.67 ± 1.40	34.27 ± 2.49	67.70 ± 11.71	27.40 ± 4.54
AUC_0−∞_	μg h/ml	12.73 ± 1.40	34.31 ± 2.49	67.76 ± 11.72	28.24 ± 4.57
MRT	h	0.46 ± 0.07	0.41 ± 0.04	0.39 ± 0.04	3.35 ± 0.23

The absolute bioavailability was calculated by comparing between oral and intravenous administration of sodium danshensu in another study. The mean plasma concentration-time curves in rats following oral administration are also illustrated in Figure 2, and the pharmacokinetic parameters are listed in Table 1. *T*_max_ after oral administration is 1.40 ± 0.30 h, indicating that the absorption of sodium danshensu from the gastrointestinal tract is in a moderate speed. The absolute oral bioavailability of sodium danshensu in rats was calculated to be 13.72%, indicating poor absorption following oral administration in rats.

### Tissue Distribution

**Figure 3:**
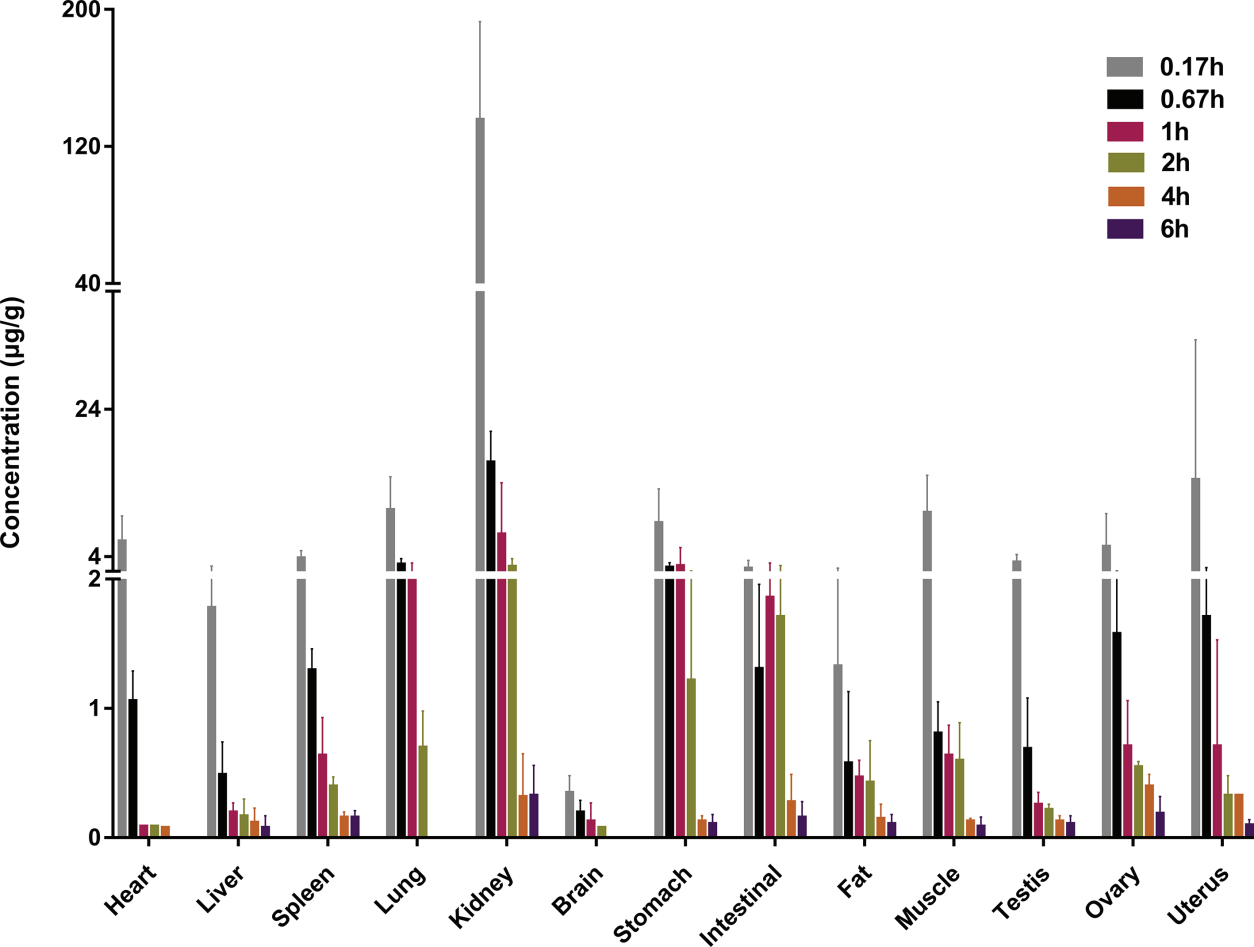
Tissue concentration of sodium danshensu at different time points after intravenous administration of 30 mg/kg in rats.

Following intravenous administration, sodium danshensu was distributed throughout the body. The concentration of sodium danshensu was detectable in all tissues studied, including heart, liver, spleen, lung, kidney, brain, stomach, intestinal, fat, muscle, testis, ovary, and uterus, as showed in Figure 3. Peak levels reached at 0.17 h in all tissues and decreased gradually in most of the tissues at 6 h pose-dose. The primary distribution site was kidney, followed by uterus, lung, muscles, and stomach. Kidney distribution of sodium danshensu is 9-fold to 388-fold higher than other tissues at 0.17 h.

### Metabolite Profiling in Rat Urine

**Figure 4:**
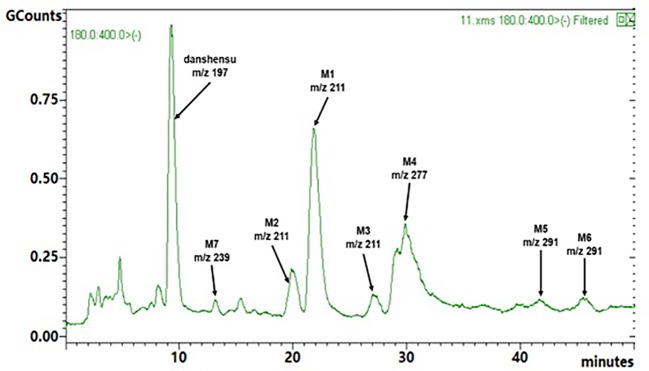
TIC of rat urine sample after intravenous administration of 30 mg/kg sodium danshensu.

**Figure 5:**
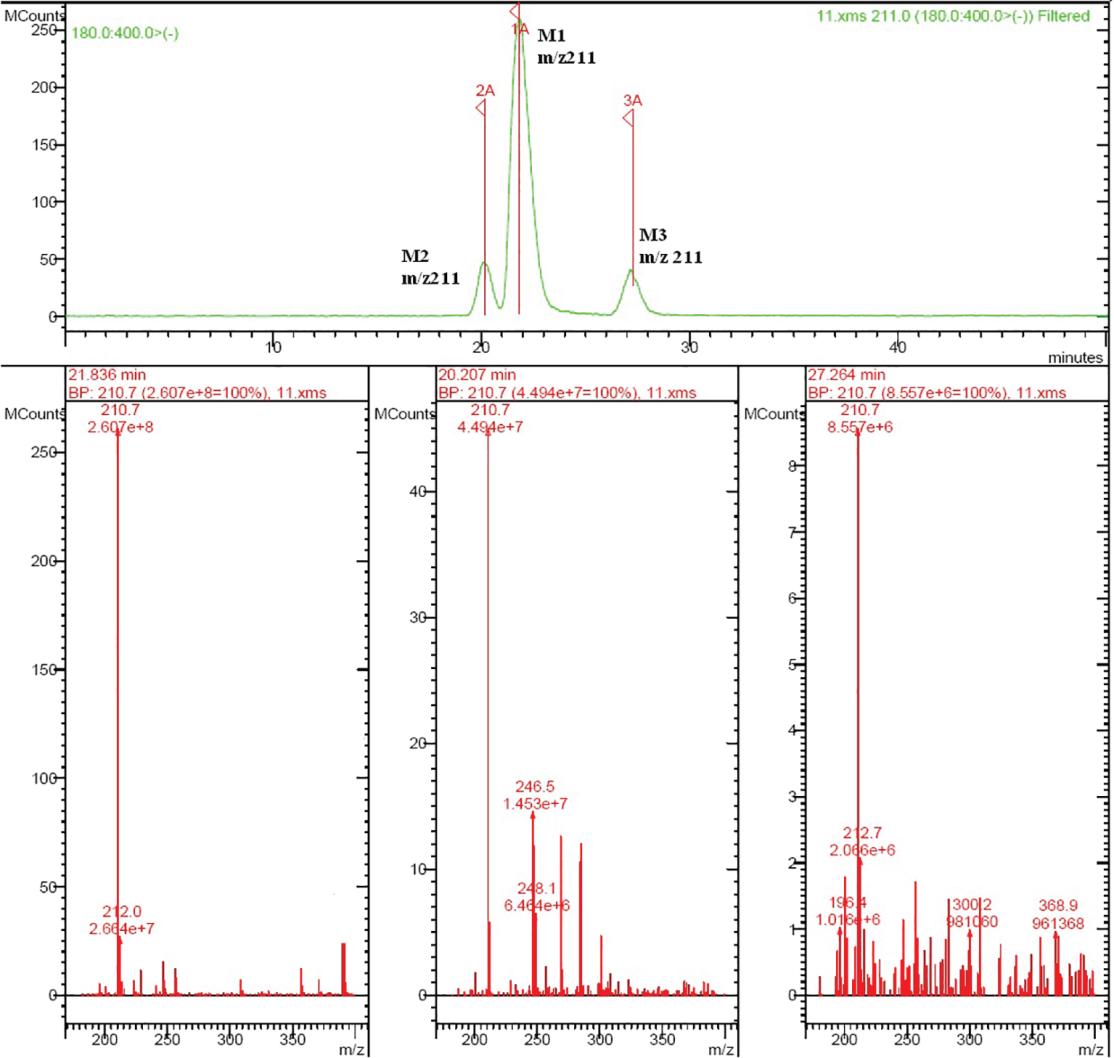
EIC of rat urine sample at m/z 211.

**Figure 6:**
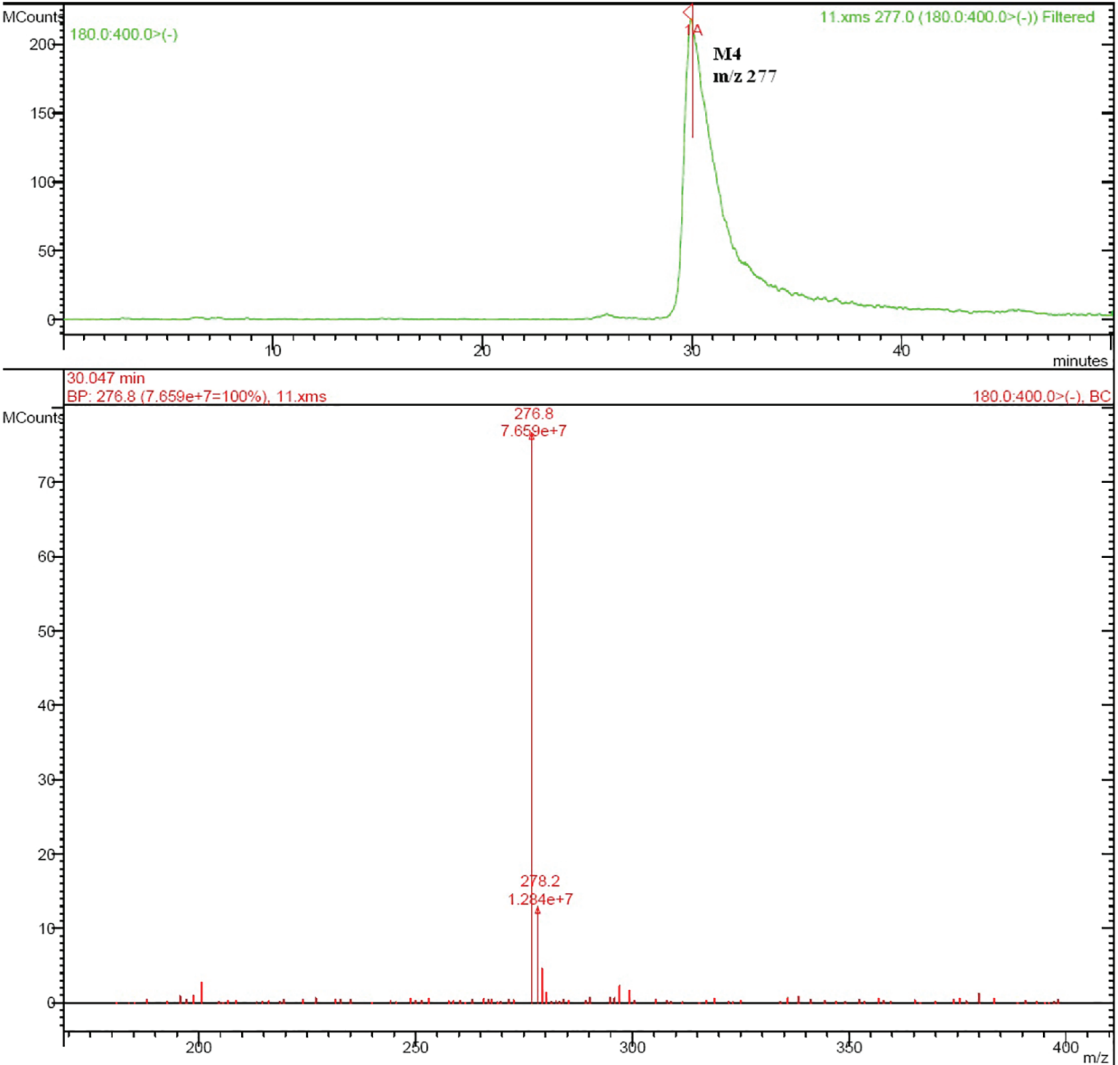
EIC of rat urine sample at m/z 277.

**Figure 7:**
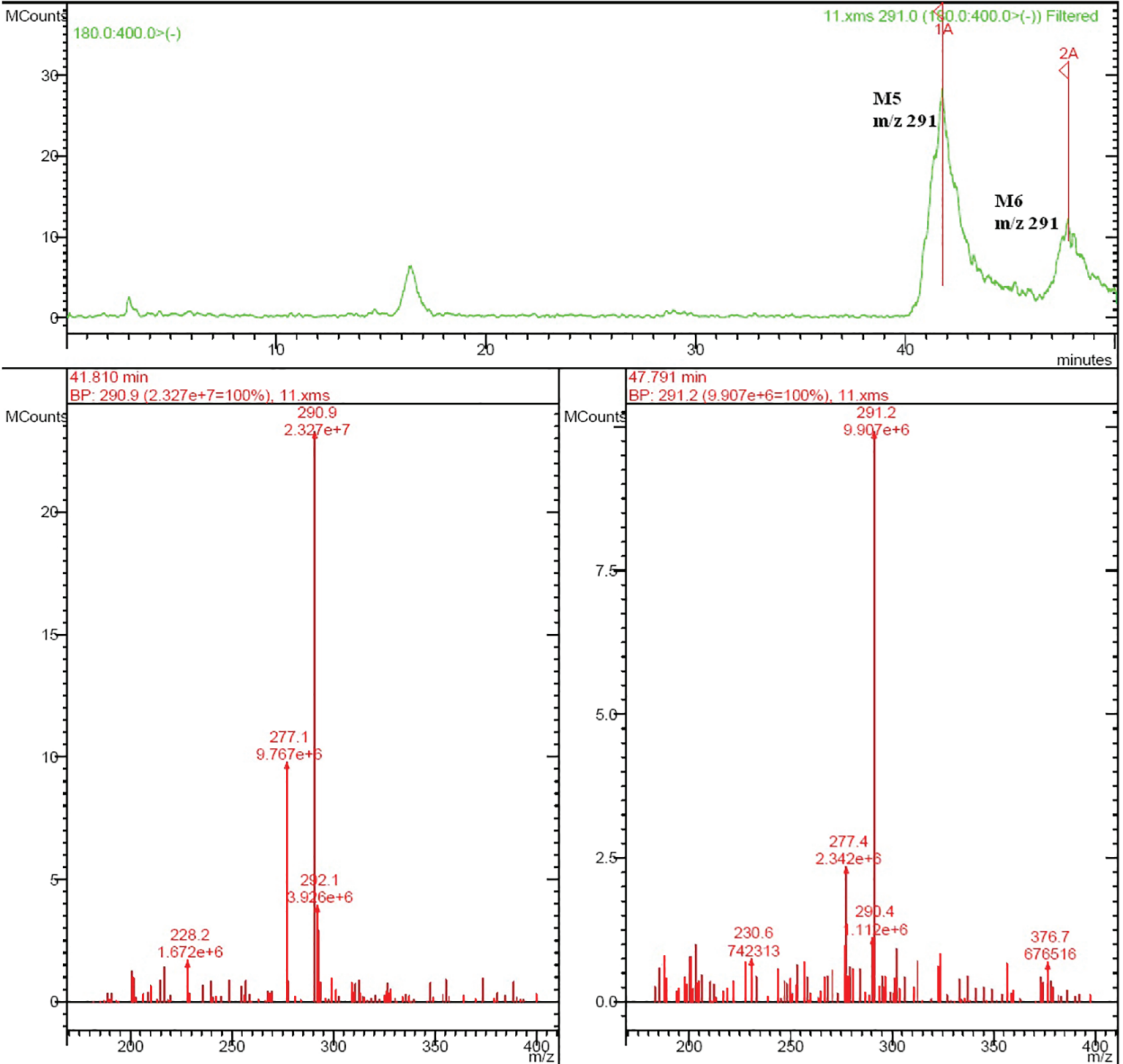
EIC of rat urine sample at m/z 291.

**Figure 8:**
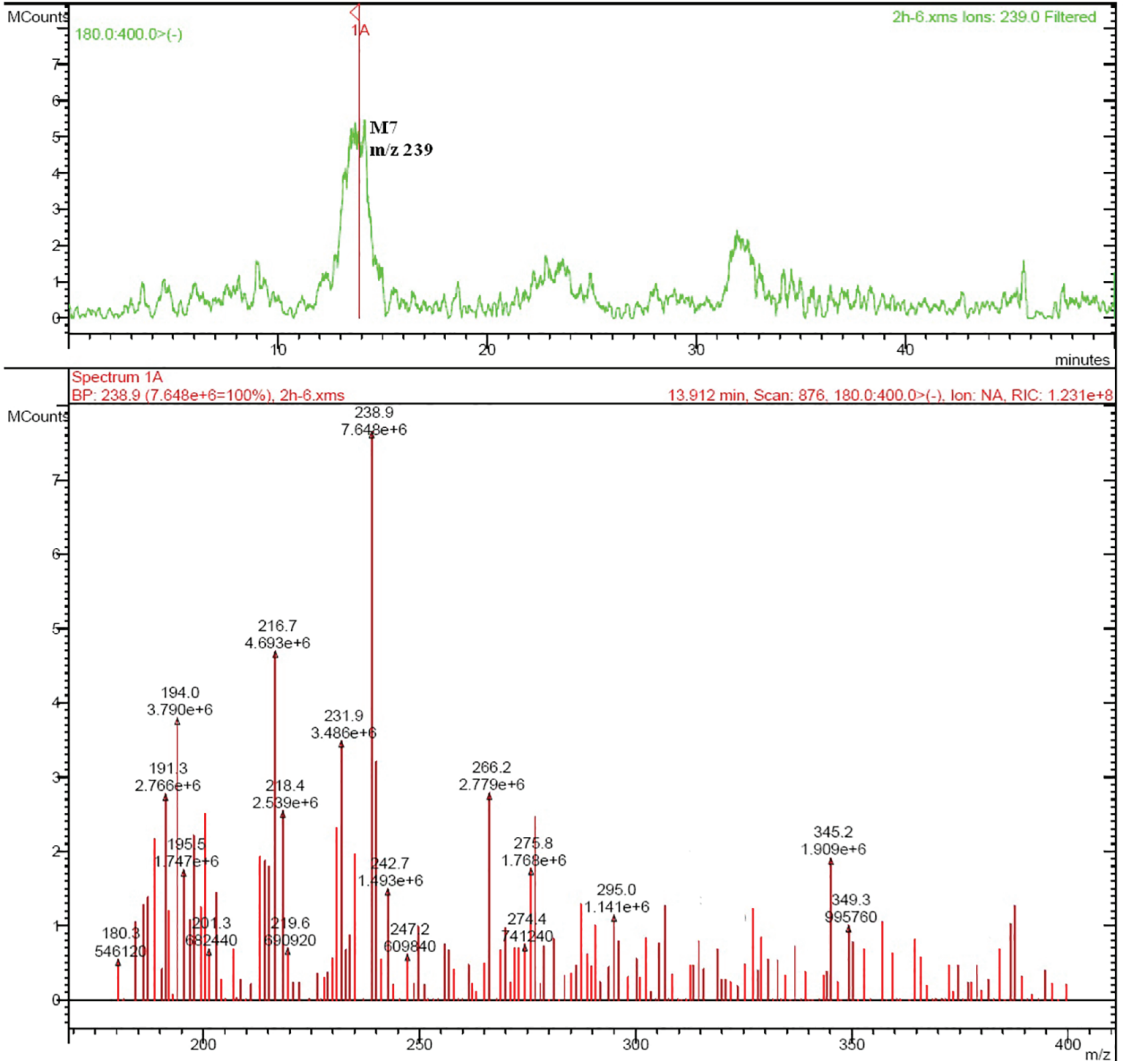
EIC of rat urine sample at m/z 239.

**Figure 9:**
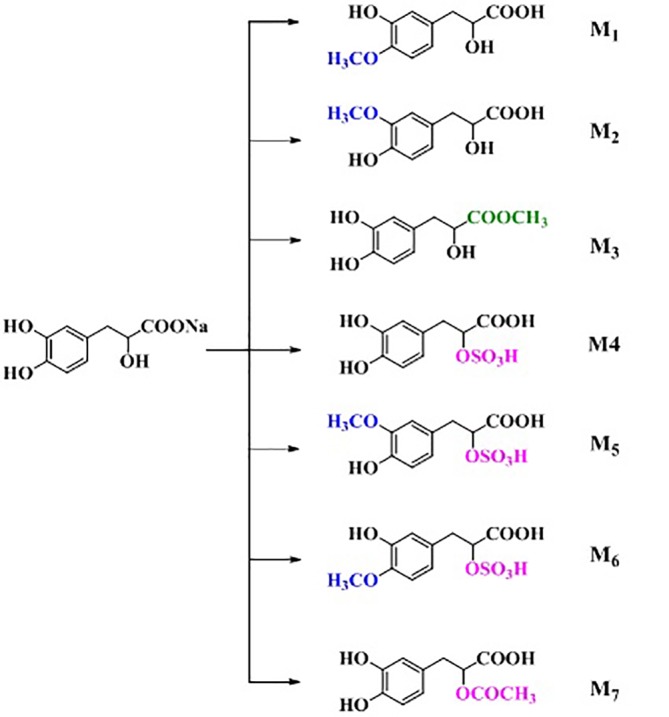
Possible metabolic pathways of sodium danshensu in rats.

Rat urine samples were collected from rats administrated with sodium danshensu and analyzed for metabolite profiling. Total ion chromatogram (TIC) of dosed rat urine was shown in Figure 4. There are seven metabolites speculated in rat urine, named as M1 to M7. Extracted ion chromatograms (EIC) of seven metabolites were displayed in Figures 5–8. M1−M3 were metabolites with molecular ions at m/z 211, 14 amu greater than that of danshensu (m/z 197). This additional evidence supported that M1−M3 were methylated metabolites of danshensu. Molecular ion of M4 was at m/z 277, 80 amu greater than that of danshensu, indicating that M4 was a sulfated metabolite of danshensu. M5 and M6 were metabolites with m/z 291, 94 amu greater than that of danshensu, presumed to be methylated and sulfated danshensu. Molecular ion of M7 was at m/z 239, 42 amu greater than that of danshensu, indicating that M7 was an acetylated metabolite of danshensu. Possible metabolic pathways of sodium danshensu in rats were shown in Figure 9.

### Excretion of Sodium Danshensu

**Figure 10:**
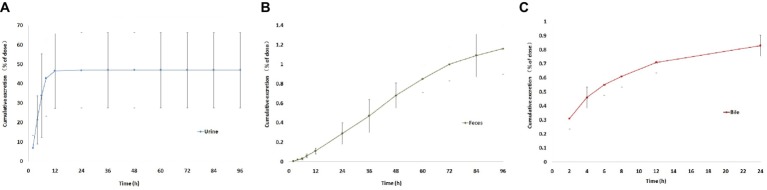
Cumulative excretion of sodium danshensu in rat after intravenous administration of 30 mg/kg. **(A)** Cumulative excretion of sodium danshensu in rat urine. **(B)** Cumulative excretion of sodium danshensu in rat feces. **(C)** Cumulative excretion of sodium danshensu in rat bile.

Excretion of sodium danshensu into urine and feces after a single intravenous administration of sodium danshensu (30 mg/kg) to rats is presented in Figure 10. Urinary recovery of total sodium danshensu for the first 12 h period is about 46.57 ± 19.25% of the administrated dose, and the value for 96 h period was 46.99 ± 19.37%. Compared with urinary excretion, feces excretion of sodium danshensu is very small, only about 1.16 ± 0.26% of the dose reclaimed in the feces within 96 h post-dose.

Biliary excretion of sodium danshensu after a single intravenous administration of sodium danshensu (30 mg/kg) is also shown in Figure 10. Total excretion into bile over 24 h is about 0.83 ± 0.11% of the administrated dose. In general, urine was the predominant route of elimination of the prototype drug, accounting for 46.99% of the administrated dose. Recovery of sodium danshensu in feces and bile were very small, with about 1.16 and 0.83% of the dose, respectively.

## Discussion

In this study, the preclinical absorption, distribution, metabolism, and excretion of sodium danshensu in rats was investigated. Sodium danshensu was poorly absorbed following a single oral administration, with an absolute bioavailability of 13.72%. It is generally known that many active compositions of Chinese Herbal Medicine have low bioavailability, which may be caused by reasons such as physical and chemical properties of drugs, damaged by intestinal environment, metabolized by intestinal microflora, metabolized by enzyme expressed in intestinal epithelial cell, efflux transported by transporters expressed in intestinal epithelial cell, and so on. Previous studies indicated that sodium danshensu was a substrate of P-gp (Yu et al., 2011). Besides, poor bioavailability, ranged from 11.09 to 28%, was also reported by other researchers (Zhou et al., 2009; Wang et al., 2015). In follow-up experiments, such as the tissue distribution, metabolites, and excretion study, sodium danshensu was given by intravenous administration.

Tissue distribution study indicated that sodium danshensu was highly distributed in the kidney. Wang reported that danshensu demonstrated a competitive inhibition towards organic anion transporters 1 and 3 (Wang and Sweet, 2013). However, no researcher has investigated whether danshensu is a substrate of organic anion transporter 1 or 3. Organic anion transporters 1 and 3 specifically expressed in the kidney mediated the renal accumulation of many drugs. Our study found that danshensu was highly accumulated in rat kidney. In addition, danshensu is an acidic compound, which is in accordance with substrate characteristics of organic anion transporter 1 or 3. It can be speculated that danshensu may be a substrate of organic anion transporter 1 or 3, which depends further investigation. From the perspective of cardiovascular pharmacological, the tissue distribution study of sodium danshensu also indicated that kidney may be the important target organs exerting pharmacodynamic effects. Preliminary research demonstrated that Danshen has an anti-hypertensive effect through the inhibition of the renin angiotensin system (Kang et al., 2002). According to the theory of traditional Chinese medicine, Danshen could promote blood circulation and dieresis by regulation the AQP2 in kidney (Dong et al., 2014). So, this tissue distribution study could provide reference for further pharmacological research.

There are seven sodium danshensu metabolites speculated in rat urine in this study, including three methyl-danshensu metabolites, one danshensu sulfate, one methyl-danshensu sulfate, and one acetyl- danshensu. Due to lack of reference substances, qualitative rather than quantitative analysis was conducted. All metabolites were deduced by their precursor ion and product ions (data not shown). Three hydroxyls exist in the structure of SAA, which may be served as methylation binding sites. Xu found that methylated metabolites of sodium danshensu displayed even higher antioxidant activity against lipid peroxidation in rat liver *in vitro*. Methylated metabolites may contribute to the pharmacological activities of SAA (Xu et al., 2014). Shen found five metabolites in rat plasma dosed with danshensu (20 mg/kg, IV), including danshensu mono-glucuronide, monomethyl-danshensu monoglucuronide, mono-methyl-danshensu, dimethyl-danshensu, and dimethyl-danshensu-monoglucuronide (Shen et al., 2009). In another study, Gu JF found five metabolites in rat urine dosed with danshensu, including two danshensu sulfate, one methyl-danshensu sulfate, one danshensu mono-glucuronide, and one monomethyl-danshensu mono-glucuronide (Gu et al., 2014).

Urinary, fecal, and bile excretion of sodium danshensu in rats after intravenous administration of sodium danshensu have not been reported yet. In this study, the majority of sodium danshensu was found to be excreted in rat urine, with approximately 46.99% of administrated dose recovered in rat urine in 96 h. Furthermore, urinary excretion of sodium danshensu was concentrated in the first 12 h, with about 46.57% of sodium danshensu, and indicated that sodium danshensu can be excreted quickly and might not result in accumulation *in vivo*. Excretion of sodium danshensu in rat feces and bile was relatively low, with about 1.16 and 0.83% of administrated dose. It means that feces and bile play minor role in the elimination of sodium danshensu.

## Conclusions

In summary, this study provides a comprehensive delineation of sodium danshensu absorption, distribution, metabolism, and elimination profiles in rats. The data demonstrates that sodium danshensu is poorly absorbed, widely distributed, bio-transformed through several metabolic pathways, and excreted mainly in rat urine.

## Ethics Statement

All pharmacokinetics studies that involved SD rats adhered to the International Guiding Principles for Biomedical Research Involving Animals, as revised by the International Council for Laboratory Animal Science (ICLAS) and the Councils for International Organizations of Medical Sciences (CIOMS) in 2012.

## Author Contributions

XM and JJ preformed the bioanalysis and prepared the manuscript. HP established the analytical method and revised the manuscript. SWa performed the animal experiment. SWa analyzed data and calculated PK parameters. GF and YL designed the whole research and interpreted results of experiments. GF reviewed the final manuscript, and all the authors have read and approved the final version.

### Conflict of Interest Statement

The handling editor declared a shared affiliation, though no other collaboration, with the authors, GF and SWa.

The remaining authors declare that the research was conducted in the absence of any commercial or financial relationships that could be construed as a potential conflict of interest.
